# Pre-K teachers’ professional identity development at community-based organizations during universal Pre-K expansion in New York City

**DOI:** 10.1186/s40723-022-00099-9

**Published:** 2022-06-07

**Authors:** Sanae Akaba, Lacey E. Peters, Eva Liang, Sherryl B. Graves

**Affiliations:** 1 Institute for Liberal Arts, Tokyo Institute of Technology, Tokyo, Japan; 2 Department of Curriculum and Teaching, Hunter College, City University of New York, New York, NY USA; 3 Research Foundation, Hunter College, City University of New York, New York, NY USA

**Keywords:** Qualitative research, Teachers’ professional identity, Universal pre-kindergarten, Preschool, New York City, Policy change

## Abstract

This study examines how policy directives and recommendations implemented during a massive universal Pre-Kindergarten expansion in New York City has impacted teachers’ professional identity. We adapted the critical ecologies of the early childhood profession by Dalli et al. (Early childhood grows up: Towards a critical ecology of the profession. In Early childhood grows up, Springer, Dordrecht, pp. 3–19, 2012) and utilized data from in-depth interviews with teachers at community-based organizations in Pre-K programs. Our thematic analysis of transcripts revealed three themes in relation to teachers’ professional identity: becoming a teacher who can play multiple roles to meet administration’s expectations is necessary; continuously modifying practice based on external support from the leadership and policymakers can be confusing; and having a brand new relationship with administrative bodies presents challenges. Data drawn from these themes reveal external factors that have influence over teachers’ professional identity. As there is heightened attention toward publicly funding early childhood in the U.S., and the need for a respected workforce, the implications of this work includes seeking out teachers’ voices to meet their localized needs to support healthy professional identity development while they adjust their practice in response to the policy change.

## Introduction

In 2014 New York City (NYC) underwent a rapid expansion of universal pre-kindergarten (UPK). This expansion resulted in significant shifts in program delivery and instructional practices to which teachers had to adapt. As teachers experienced the rapid changes brought on by UPK expansion, the authors, as early childhood educators and researchers, examined how various factors including time, place, personal backgrounds and experiences, engagement with policy, and programmatic recommendations shaped their professional identity. The early childhood field has seen a high turnover rate among early childhood teachers/caregivers as they are often undercompensated, and overworked, while receiving little to no benefits. UPK teaching now has to align with kindergarten readiness, and this heightened focus on long-term school success has been adding pressure to their already important work. In order to change this and to help teachers feel more supported, we, as researchers, practitioners, and policymakers, in early childhood education, need to listen to them. We also need to pay attention to how decisions made for or about them may impact their work.

Teachers’ professional identity has been recognized as a key component in understanding their professional lives, such as quality of teaching, motivation, well-being, commitment, and resilience (Day & Gu, 2007; Day et al., 2007; Hong, 2010). Teachers’ professional identity is influenced by their past experiences and obtained knowledge that significantly shapes their beliefs, skillset, motivation, decision-making, behaviors, and actions in their professional roles. Professional identity is constructed and maintained through the interactions between socio-cultural factors and individual teachers’ internal and psychological processes (Hong et al., 2017) in various social contexts such as race, gender, social class, culture, language, immigrant status, and country of origin. Professional identity, therefore, is considered to be individually unique. Furthermore, identity develops over time through the interpretation and re-interpretation of social interactions, such as interpretation of messages from the leadership and policymakers, and is not fixed or static (Hong et al., 2017, p. 84–85).

The field of early childhood care and education (ECCE) has garnered greater attention as years of research across disciplines has revealed the benefits of early care and education for later school success, as well as economic growth and development (Yoshikawa et. al., 2013). Consequently, there is a notable increase in the amount of funding going towards public preschool programs. NYC implemented its UPK preschool expansion, Pre-K for All (PKFA), in 2014, as one of the primary political initiatives of Mayor Bill de Blasio. PKFA provides access to free, full-day, high-quality early childhood education to all 4-year-olds in NYC, regardless of the socio-economic status (NYC Department of Education, 2019). The UPK expansion was rolled out rather rapidly, as the approximately 19,500 available full-day seats in 2013 significantly increased to over 70,000 in 2016 in both public school and local community-based organization (CBO) settings. According to Hustedt et. al. (2018), teachers are primary actors and enactors of early childhood care and education; therefore, it is crucial to observe and understand their daily experiences through their policy enactment, and how that influences their values, beliefs, and needs that affect the development of their professional identity.

As many researchers argue, teachers’ professional identity is at the core of the teaching profession because it is influenced and shaped by teachers’ other identities and daily experiences within various contexts, and how teachers interpret and reinterpret professional identities through socialization and self-reflection. However, little attention has been paid to teachers’ perspectives and experiences, especially the variability in teachers’ experiences and how the shifting landscape of Pre-K education has been affecting the development of teachers’ professional identity since the UPK expansion in NYC. Our aim is to shed light on their experiences to provide people outside the classroom a more in-depth understanding of the ways in which policies shape teachers’ daily lived experiences. Utilizing the three entities of teacher professional identity, multiplicity, discontinuity, and social nature (Akkerman & Meijir, 2011), as our framework, we examine whether the factors during policy changes have impacted how teachers shape and reshape their professional identity.

## Literature review

The United States has experienced a significant increase in publicly funded preschool programs in recent years, specifically for children who are 3 to 5 years old. The significant push for early childhood education policy reform has drawn much attention by policymakers and caregivers, and the discourse around early childhood education centers on how to establish more equitable experiences for young children and their families (Zigler et al., 2006). What is more, the movement to expand public preschool programming bolsters efforts to align early childhood education with the K-12 system of schooling. Years of education research and recent neuroscience research has supported that early learning does matter for students’ future outcomes such as high school graduation, college entrance, involvement in criminal activities, and physical and emotional health over all (Shonkoff et al., 2012). The success in promoting such publicly funded, universal early childhood education experiences from some states, such as Florida, Oklahoma, and Georgia (Gormley et al., 2005, 2018) that had started publicly funded early childhood education attracted other states to implement public state-level or federal-level funded Pre-K programs over the past 10 years resulting in rapid shifts toward the trend toward publicly funded early childhood education. These shifts have impacted preschool teachers in numerous ways, and changes they experience range from professionalization and new requirements for certification to curriculum, teaching, and assessment.

Early childhood educators struggle with recognition and development of their professional identity as their profession has been often considered ‘glorified babysitting’ that requires only a caring nature and maternal instinct (Share et al., 2011; Shpancer et al., 2008; Tuominen, 2003). This devalued image of early childhood educators has marginalized them in the education field and the society, which can negatively impact the construction and development of identity as care providers and educators of young children. For example, in Tuominen’s study in 2008, Ms. Burbank, an African American provider, asserts she dislikes “the condescending attitude toward the child care profession. The public doesn’t understand the significance of our work. It is demeaning. You feel belittled” (Tuominen, 2008, p. 148).

Recent research suggests that teachers’ professional identity, well-being and mental health can be greatly impacted by bureaucratic changes at policy and management levels such as credentialing, professional development (PD), curriculum, workload, standards, quality control, and expectations for student outcomes (Skinner et al., 2018). When such changes happen rapidly, teachers’ work experience may be a source of distress, frustration, unhappiness, and dissatisfaction (Douglass, 2017).

### Teachers’ professional identity

A growing body of research has been done on early childhood teachers’ professional identity and broadened the discourse about teaching practice and professionalism in the field (e.g., Dalli et al., 2012; Harwood & Tukonic, 2017). Dalli et. al. (2012) argue that professionalism in early childhood practice has multiple layers of influence that teachers carry from their social contexts, and cannot be simply defined through lists of professional qualifications and individual attributes. The contributing factors to the construction and development of professional identity include teachers’ perceptions of their values within their profession and community, personal qualities, relationships, status, and qualification (Dalli, 2008; Tucker, 2004). For example, Thomas (2012) suggests that early childhood educators typically have two types of relationships: one with parents or caregivers and the other with colleagues. In addition to these relationships, given the heightened attention to publicly funded preschool education, a number of ECCE teachers are experiencing the added relationship with the local and/or state government. ECCE teachers are also expected to increase parent participation, parent education, and parent empowerment (Thomas, 2012). Furthermore, a sense of belonging to a community and workplace and acceptance by supervisors and peers are crucial factors that also shape professional identity (Sachs, 2003). The societal normalization that kindergarten has become the new first grade (Bassok et al., 2016; Miller & Almon, 2009), has shifted public expectations for a heightened academic focus in Pre-K classrooms. Therefore, ECCE teachers are reconceptualizing their understanding(s) of kindergarten readiness and Pre-K education, while maintaining their personal values and beliefs, and being adaptive to culture and policy related changes (Sachs, 2001).

The professional identity of teachers involves an ongoing process of construction based on experiences in time, contexts and relationships, as opposed to being fixed and stable. Akkerman and Meijir (2011) show how research generally contextualizes teachers’ professional identity within or across three entities: multiplicity of identity, ‘sub-identities’ that teachers develop in their profession; discontinuity of identity, an ongoing process of construction; and social nature of identity, identity associated with various social contexts and relationships.

Multiplicity refers to the multiple identities that teachers may embody. Day et. al. (2007) argues that teachers tend to hold three dimensions of identity: professional identity, situated identity, and personal identity. Beijaard et. al. (2004) argue various contextual factors, such as school community, and relationships with other school staff and colleagues, and students’ families, influence teachers’ sub-identities. More recently, researchers generally describe teachers’ professional identity to be one component of the multiple perspectives that comprise an individual teacher’s identity (Sutherland et al., 2010). Similar to other aspects of identity, an individual’s professional identity is situated in their position within the society, their socializations and interactions with others in various social and cultural contexts and their interpretations of such experiences (Gee, 2000; Geijsel & Meijers, 2005).

Discontinuity refers to the idea that identity continuously shifts from moment to moment in various contexts. For example, Rodgers and Scott (2008) define professional identity as “evolving, consciously and unconsciously constructing and being constructed, reconstructing, and being reconstructed in interaction with cultural contexts, institutions, and people” (p. 715). Furthermore, Beijaard et. al. (2004) describe that identity development is an ongoing process in which teachers interpret and reinterpret their experiences in different contexts. Thus, a teacher’s professional identity is not a standpoint of their teaching practice that they develop once and is fixed throughout their professional career.

Lastly, social nature generally refers to the idea that identity is contextual and constructed based on interactions and relationships that teachers hold. Rodgers and Scott (2008) argue that contexts naturally shape teachers’ perceptions of who they are and how they are recognized by others. In her focus group study, Cohen (2010) discusses how interactions with colleagues can influence each other to form their own professional identity and how this identity is a dynamic process constituted through daily interactions with colleagues. A teacher’s professional identity is influenced and shaped through their socialization within the contexts that they live, such as professional community, friendship, family, culture, and the larger society.

### Teachers’ experiences and professional identity development during policy change

As reviewed above, research on teachers’ professional identity describes identity as fluid shifts in various contexts and relationships. Some components such as increased needs and opportunities in credentialing in ECCE and PD are crucial in supporting the key players of policy enactment and understanding how teachers are able to respond to policy changes and demands (Brown & Englehardt, 2016). As public investment in ECCE and UPK increases, the requirements on teacher credentials have also changed state by state (Gomez et al., 2015). In NYC, for example, PKFA lead teachers are required to be on a study plan and obtain a Master’s degree and NYS teaching credentials within 3 years (Reid et al., 2018). While most public school teachers already hold such credentials, a number of CBO teachers have had to go through the credentialing process on top of their daily teaching workload.

Professional learning is also essential for early childhood educators’ professional growth in order for them to understand and reflect on their teaching and their students’ learning (Brown & Weber, 2016). It is, therefore, important that all teachers are provided with PD opportunities and able to participate in them to shape their professional identities in the midst of policy changes. Wilinski (2017), however, describes the unequal variability of access to opportunities for PD experiences based on school settings (public district school or community-based childcare center) due to inflexibility caused by program scheduling and low adult coverage, as a result of Wisconsin’s statewide universal Pre-K program policy. While teachers who are not provided PD opportunities fail to learn about policy reform and recommended practices that are directly related to accountability, they are also excluded from their professional communities that might help them develop stronger relationships with their peers and colleagues (Akaba et al, 2020a). Therefore, Pre-K teachers and administrators at CBOs experience the need to consistently adapt their identities and expectations to navigate the public Pre-K system so that they meet the requirements of the Pre-K policy. Therefore for this study, we focus on Pre-K teachers at CBOs to investigate how implementation of the UPK expansion policy influences teachers’ daily lives and ultimately their professional identity in NYC.

### Brief background of recent NYC’s early childhood care and education

In 2012, NYC’s Bloomberg administration launched the early childhood care and education system for children aged zero to five, EarlyLearn,^1^ through which the city’s child care, Head Start, and UPK programs were funded. This complex funding system involved the Administration for Children’s Services (ACS) for childcare and Head Start, and the DOE for UPK (Gelatt, 2014). Moreover, public school Pre-K teachers were provided with 4 days of DOE sponsored PD opportunities, while CBO teachers were provided with 10 days of on-site PD opportunities hosted by their own sites throughout a school year. In July 2019, the EarlyLearn system was merged into the DOE to provide early childhood services for all children up to age five. This was a significant reform for the DOE to aim at more equitable distribution of resources and funding to all early childhood programs and services.

The 2-year-plan for expansion of access to UPK programs for all 4-year-olds in all five boroughs in the city, regardless of family income, was one of the primary campaign promises of Mayor Bill De Blasio when he took the mayoral office and control in January 2014. During the initial expansion in 2014–2015, CBOs experienced a significant increase in full-day seats of approximately 3000 to 31,000 (NYC Independent Budget Office, 2015). Thus, in the first 2 years of expansion there was a steep increase in the number of new Pre-K teachers recruited for Pre-K programs at CBOs. While the expansion has increased accessibility to early education for a significant number of children and families in the city, the initiative may need adjustments to better support teachers and site leaders in CBOs that are less experienced with the leadership of the DOE (Reid et al., 2018).

The complex funding streams established through EarlyLearn were sustained, and monitored by different auspices such as ACS and DOE^2^ (Reid et al., 2018). Therefore, the beginning of PKFA was the very first experience of complicated partnerships with the DOE and ACS for the majority of Pre-K teachers and administrators at CBOs. For example, added public funding required heightened accountability through assessments and evaluations, which added to teachers’ workloads. This also influenced quality improvement efforts—the DOE’s social workers and instructional coordinators were assigned to the majority of the PKFA programs, and conducted site visits for additional support and coaching since 2014 (Reid et al., 2018). As the PKFA program was rolled out universally, these efforts diminished the focus on CBO teachers’ localized needs (Akaba et al, 2020b; Michael Luna & Grey, 2019).

In their study on PKFA programming and implementation, Reid et. al. (2018) describe how the Pre-K programs looked differently in public school and CBO settings in NYC. For example, Pre-K teachers in public schools are 50% White, while more than 70% of the teachers in CBOs are people of color. Early childhood teachers in local community organizations, that have historically served the communities in need, are predominantly people of color.

Teaching credentials held by Pre-K teachers in public schools and CBOs varies (Reid et al., 2018). Reid et. al. (2018) found that 100% of the public school teachers held a Master’s degree^3^ compared to 66% of the CBO Pre-K teachers. Even with the same credentials, the average teacher salaries differed by nearly $30,000 ($73,471 in public schools versus $43,660 in CBOs), which translates into $1146 weekly or $38 hourly difference.^4^ In addition, public school teachers receive more comprehensive benefits. Consequently, such disparities in salaries and benefits have resulted in movement of teachers from CBOs to public schools (Reid et al., 2018). In July 2019, Mayor de Blasio and the NYC Day Care Council announced a new plan to close compensation gaps for Pre-K teachers at CBOs. Under this plan, there is an approximately $20,000 increase for certified Pre-K teachers with Master’s degree and $17,000 increase for certified Pre-K teachers with Bachelor’s degree by October 2021.

### Conceptual framework

We utilize the conception of teachers’ professional identity suggested by Akkerman and Meijir (2011) as our conceptual framework. Furthermore, in order to understand how the teachers’ professional identity is affected during the policy change in the education systems, we also utilize the understanding of ecology of early childhood profession. In their study on behaviors of early childhood teachers, Dalli et. al. (2012) adapted the ecology of human development systems theory (Bronfenbrenner, 1979; Garbarino & Abramowitz, 1992; Urban & Dalli, 2012), and borrowed the definitions of ecological systems in early childhood profession illustrated in Table 1.

**Table 1:** Definitions of ecological system terms as used in this paper. Adapted from Dalli et. al. (2012, p. 7)

Ecological level	Definition	Examples
Microsystem	Situations in which the practitioner is physically present and has face-to-face contact with influential others	Children Co-workers Caregivers and families Social worker, etc.
Mesosystem	Relationships between the *Microsystems*; connections between situations	Team connections Multi-/inter-professional work such as children and caregivers, families and site leaders, and families and a social worker
Exosystem	Settings in which practitioners do not participate but in which significant decisions affecting them are made	Local/regional body authority Parents’ workplace, such as the DOE and Early Learn in NYC
Macrosystem	‘Blueprints’ for a particular society; assumptions about ‘how things should be done’	Values, shared assumptions, broad ideological patterns of a particular culture; socio-economic and political context, such as domestic and international research organizations including OECD
Chronosystem	Developments of the ecological system over time	Socio-historical context such as racism, long-held feminized profession of ECCE, Covid-19 pandemic

**Fig. 1:**
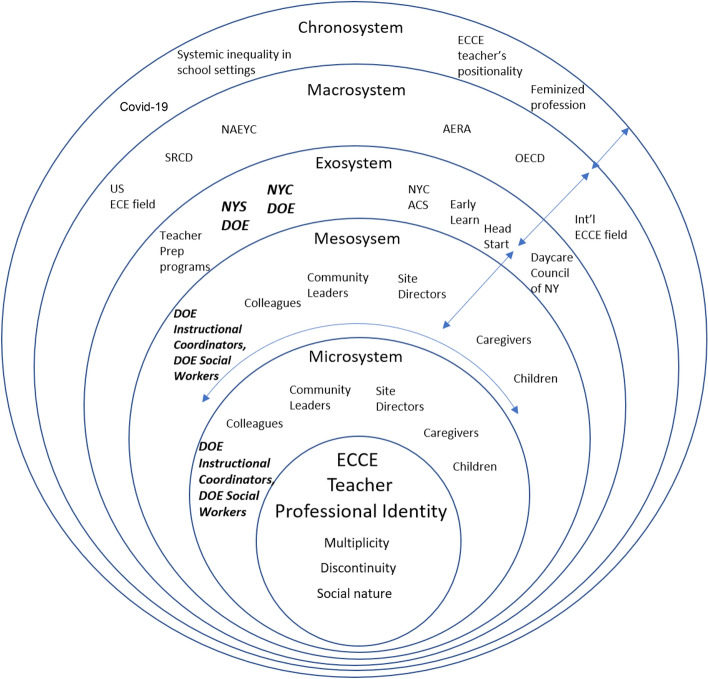
Ecological system of ECCE teacher professional identity at NYC CBOs

Our primary interests center around how the context of Pre-K expansion and the fostering of new relationships with other entities (e.g., CBOs collaboration with DOE) through the policy implementation process has influenced teacher professional identity: multiplicity, discontinuity, and social nature as outcomes of new policy implementation. Additionally, our focus is centered on how teachers’ professional identity is influenced by factors within this ecological system of the early childhood profession during a period of rapid policy change. We, therefore, developed our own framework based on Akkerman and Meijir’s professional identity framework (2011) and Urban & Dalli’s ecology of early childhood education professionals (2012). We call this the Ecological System of Teacher Professional Identity (see Fig. 1).

In this framework, every layer of the system influences a teacher’s professional identity. Microsystem refers to those who individual teachers have relationships and work with closely, such as site directors, caregivers, children, community leaders, colleagues, and coaches, and social workers. Mesosystem refers to the interactions and relationships among the actors in teachers’ Microsystem, which indirectly influence teachers’ professional identity. Exosystem is typically where teachers work and where the funds are from, such as the local and state DOE, NYC Administration for Children’s Services. Macrosystem is namely the societal norms and dominant ideas about the ECCE professions and field are created by domestic and international organizations such as the National Association of Education for Young Children, American Educational Research Association, Society for Research in Child Development, and the Organization for Economic Co-operation and Development. These organizations create norms and expectations for the profession and therefore have great impacts on decision-making within the federal, state, and local agencies that develop policies and budgets at the Exosystem level. Chronosystem refers to a socio-historical context of ECCE in the U.S. and abroad, including the feminized and often racialized profession of the ECCE, teachers’ positionality and the systemic inequality seen in various school settings. Seen in italicized bold are the primary entities that brought on significant changes to the CBO teachers since PKFA implementation started. Since December 2019, Covid-19 pandemic has impacted the daily lives of people around the world. It is the beginning of the 3rd year of Covid-19, and we believe the pandemic has become part of teachers’ chronosystem that influences their exosystem, and reaches down to the microsystem level within the ecologies of their professions. For example, the spread/rise of COVID-19 cases in March 2020 forced a shutdown in the NYC public school system including PKFA programs. By summer 2020, caregivers of young children reported their struggles working remotely while taking care of their young children due to a lack of childcare. Childcare centers at CBOs were the first ones to re-open despite much opposition from teachers and administrators. Although we collected our data pre-Covid, we aim to highlight the changes across all the systems interacting with each other and which eventually have influence on teachers’ professional identity. The tensions and possibilities with regard to fostering teachers’ professional development that surfaced during the course of our study in the years prior to the global pandemic continue to bear relevance. Further, many of the issues that are faced by early care and education professionals are long-standing, but are being exacerbated by the current struggles brought on by the global health crisis and social and political turmoil.

## Methods

Data presented in this paper are part of a phenomenological study which was part of a larger qualitative research project on the experiences of PKFA teachers that occurred during 2016–2017. Provided the possible disparities reviewed in the previous section that NYC PreK teachers might have experienced, we decided to focus on the experiences of CBO teachers to deepen our understanding of the factors that are shaping their professional identity when the implementation of PKFA was their first time working within the NYC DOE system for most of the teachers at CBOs. Our research team consisted of 10 members, one of whom was a native speaker of Spanish, another of Mandarin Chinese and Cantonese, and the other was fluent in Bengali. Four of the team members had expertise and teaching experiences in early childhood education and three in education and teaching, the other four team members had experience in fields related to early care and education (e.g., psychology). We recruited eight PKFA programs in three of the five boroughs of NYC based on the sample provided by MDRC, an education and social policy research organization, which included programs in low, moderate, to high resourced communities.^5^ We cold called, emailed, and visited the programs on our list and met with site leadership to provide information about our study and to invite them to participate.

PKFA programs at seven CBO sites agreed to be a part of this study, and a total of twelve Pre-K lead teachers were recruited for their participation in this study (see Table 2 for demographics and descriptions of teachers). Those teachers, who happened to be predominantly Black^6^ and Latina,^7^ immigrants, or immigrant descendants, and these teachers served children of color, completed a demographic survey and participated in a series of 5 monthly interviews on their experiences in Pre-K programs. The research team members conducted a total of five in-depth, semi-structured interviews with each of the twelve teachers. Five teachers, however, needed to have combined sessions due to time constraints. Each interview session was approximately 60 min, audio recorded, and each participant was assigned a unique identification code. Interview questions were developed to understand teachers’ professional identity based on their perspectives on their practice and the level of agency that they thought they had as a professional in their planning, teaching, and decision-making. The interview questions were formed around their choices in curriculum and assessment method, types and frequency of family engagement, the types of support available for them, and the heightened interests in kindergarten readiness in preschool programs. Examples of questions asked include: *What has changed since Pre-K for All started in terms of your teaching practice, choice of curriculum and assessment, and decision-making? What curriculum do you use to plan learning experiences for the children in your class and how did you decide on using it? How do you document children’s progress, and how frequently do you it? How did you learn about the DOEs units of study, and do you use it? What are some ways you communicate with parents? How do you help children get ready for kindergarten? What recommendations would you share with the DOE about improving Pre-K for All?* These questions helped us understand teachers’ daily experiences pre- and post-PKFA which might have substantially influenced professional identities of our CBO teachers.

**Table 2:** Characteristics of participants

Participant	Age	Self-identified race and/or ethnicity	Years of teaching experience
Ms Frances	36–45	Black	20
Ms Candice	26–35	Black	6
Ms Felicia	46–55	Black	24
Ms Darlene	46–55	Black	21
Ms Kimberly	26–35	Black	5
Ms Jessica	26–35	White	2
Ms Stephanie	26–35	Latina	3
Ms Heather	18–25	White	3
Ms Samantha	26–35	Mixed race, Black	2
Ms Alicia	36–45	Black	3
Ms Mabel	55+	Black and Latina	20
Ms Gloria	46–55	Black	< 1

Data were analyzed utilizing a combination of deductive and inductive coding methods and the 6-step thematic analysis method proposed by Braun and Clarke (2006). First, recorded interviews were transcribed, and researchers read and reread the transcripts multiple times to familiarize themselves with the data. Second, we analyzed the data deductively based on the initial codes that were related to teachers’ daily experiences influenced by the changes since the beginning of the PKFA and our framework. Codes were generated prior to analysis because our conversations with teachers revealed possible themes, ideas, or understandings of their daily experiences. Initial codes fell under the categories of PD, assessment, internal and external demands, children and family, curriculum, to name a few. Third, after the initial analysis, an inductive analysis was conducted using ATLAS.ti 8.1. by utilizing a set of codes that reflected the researchers’ immersive experiences in the CBO classrooms in the data. We reread the coded texts and checked for interrater reliability on coded data among the researchers to ensure there were at least 80% agreement. Each transcript had at least two coders. Fourth, we generated a set of themes that reflected teachers’ professional identity influenced by their daily experiences in the PKFA programs. Some of the themes included new relationships with DOE, opportunities for PD, assessment data collection and management, family engagement, kindergarten readiness, new DOE recommended practices, and demands on teachers’ time. Fifth, we reviewed the themes, and we defined and refined the specific meaning of each theme in relation to the data. Lastly, we sorted out excerpts and selected examples that were compelling to answer the research questions. Though the data coded were collected as part of a phenomenology study, not specifically on teachers’ professional identity, a good number of responses were closely related and coded as teachers’ professional identity. We, therefore, decided to report the following findings.

## Findings

### Pre-K for All policy implementation at classroom level

Our initial dialogues with the CBO teachers were about how the Pre-K expansion policy impacted their classroom practices, that is, how changes in teachers’ exosystem impacted their practices in the microsystem. The CBO teachers described how their workload had changed (e.g., added requirements, shifting expectations, and heightened accountability). The DOE instructed the teachers to follow the New York State Prekindergarten Foundations for Common Core (2014)^8^ as instructional standards and utilize Units of Study, an interdisciplinary curriculum consisting of ten project-based units generated by the Division of Early Childhood Education. The DOE also sets three deadlines for authentic assessment checkpoints every school year by which teachers are required to input and submit collected data of learning progress of each child in the classroom. In the 2016–2017 school year, teachers were also required to attend 4 days of PD workshops. ECERS (Early Childhood Environmental Rating Scale: Harms et al., 1998) and CLASS (Classroom Assessment Scoring System: Pianta et al., 2008) evaluations are conducted regularly, and the scores are posted as program quality snapshot online (https://tools.nycenet.edu/snapshot/2021/). Expectations for family engagement also changed to promote more reflective and reciprocal partnerships. Instructional coordinators and social workers were assigned to each program to support teachers around curriculum and instruction, environmental planning, social-emotional and behavioral strategies, and so on, all of which impacted teachers’ lives at their mesosystem and microsystem level. Throughout the next sections, we present the themes that emerged in our analysis around these shifts.

### Theme 1: Becoming a teacher who can play multiple roles to meet DOE’s expectations is necessary (multiplicity)

The teachers in our study generally described their experiences adjusting their values, beliefs, and needs to DOE’s new added requirements and shifting expectations since the Pre-K expansion at teachers’ exosystem level. As such, their responsibilities changed which in turn had influence over their professional identity in order to fulfill the obligations set by the DOE and their instructional leaders. One of our teachers expressed it as “doing it in the DOE’s way.” For instance, one added requirement was utilizing an authentic assessment system to monitor children’s development and inform teaching practice. It could take several days to input assessment data into the online system for every child in the class. Ms. Stephanie described the need to allocate her weekend time in order to complete the assessment by the deadline: “Most of the time I do it on the weekends at home. Because there is really very little time at work in the classroom…”.

While the DOE’s reason to require teachers to conduct authentic assessment is well intentioned—to help teachers assess their own teaching practice and individualize their instructional strategies for children at various developmental stages—teachers did not always interpret the purpose of assessment in this way. For example, Ms. Jessica illustrated how she would explain the purpose of authentic assessment to parents by stating:

I don’t really approach parents about [the assessment], and most of them don’t know about it. They don’t know anything about it. But if I do, I just explain that it’s the DOE’s way of tracking the kids to make sure that they’re developmentally appropriate and they’re meeting standards, and it’s like the DOE’s way of keeping track of the kids.

In another interview, Ms. Jessica also elaborated on how her practice needed to shift to “doing it the DOE way”. As a teacher in a Pre-K program at a CBO, this shift in her exosystem was an extremely significant change for her practice and roles at her microsystem level:

I just got a whole new way that we’re doing lesson plans. I was just in an hour meeting and we were given, we’re doing it the DOE way. So that’s different for us and that’s a lot more work. Constantly writing reports. Authentic Assessment… I feel like it is a big demand and it’s time consuming, it’s super time consuming. I’ll probably do it this weekend. Other times we’re, we’re prepping for the next day. So if we’re prepping for the next day, I can’t write a report. So it’s kind of all of it. It’s all really time consuming.

Many of our teachers also played or tried to play a role as parent educator or supporter in the form of family engagement at their microsystem level, which is common practice and is also required by the DOE, in another word, teachers’ exosystem. Some teachers prepared materials for family engagement and planned events in addition to the daily operation of their programs (e.g., lesson planning, curriculum building, and data collection for assessment). Teachers also expressed the need to increase family engagement that encourages effective community within a Pre-K site, which is also one of the DOE’s expectations. Ms. Samantha prepared a weekly “home activity” for the families who needed guidance in supporting their child’s early education, which indicates that the shift in her exosystem caused changes in the relationships with parents in her microsystems.

Spring Break... If I don’t send home a little home activity, I am pretty sure they don’t do anything. I tell them I don’t send activities every day, but you can go get little coloring books. Don’t wait for me. They don’t have to depend on me. You know, if I send something home to reinforce what I am doing in the classroom…. I am happy to send activities, but it’s like extra paperwork for me. I am not really required to get parents homework, you know, for PreK. You know, I give it to them because I know it will help parents and my students. It reinforces everything.

We also saw similar examples in our other study on homework in Pre-K classrooms. We found in the study that some teachers felt pressured to give young children homework (or home activities) because of the heightened expectations for family engagement and kindergarten readiness from the DOE (Authors b, 2020). There were even demands from families for homework. Some teachers also thought this would help children retain classroom learning over the winter and spring breaks, and even the summer break before kindergarten. That is, the exosystem level changes impacted teachers’ roles at their microsystem level as well.

CBO teachers described that they felt playing multiple new roles, not only as teachers, but also as photographers, data collectors, parent educators, and so on, to meet DOE’s expectations was necessary. Their professional identity consisted of multiple identities that they may have embodied. Especially since Covid-19 pandemic started and CBO sites were required to reopen in summer 2020, additional roles had been added to their identity—a mental and physical health manager and tech-savvy facilitator for both their remote and in-person classes. The CBO teachers worked under multiple agencies including DOE and ACS, that may or may not align at the policy level, which also amplified their experience of “wearing multiple hats,” which related to the multiplicity of teachers’ professional identity.

### Theme 2: Continuously modifying practice based on external support from the DOE is confusing (discontinuity)

As a form of support for teachers, the DOE has generated and provided online resources called Units of Study (New York City Department of Education, 2015), which include ten units with sample weekly lesson plans and activities. When asked to explain how she decided to use the Units of Study, one teacher said, *“All Pre-K programs have to comply with the DOE, and that’s what I do (although I had curriculum that I used before)”.* Ms. Frances and Ms. Felicia also noted, respectively:

In the computer you can see the things you have to do weekly [in the Units]. So that’s what I do. I go to the computer, I check it every week, I do what I am told to…

I think we have to focus on that Units of Study because when they [DOE personnel] come, that’s what they’re looking for. You want to get a check, check in the sense of good job, even if you don’t agree with it. Give them what they want, right?

These teachers seemingly had to comply with their understanding of DOE’s standards and expectations by utilizing the Units of Study, even when their Pre-K programs used other curricula before the expansion started; that is, teachers felt in need of constantly constructing and reconstructing part of their identity as an early childhood educator (Rodgers & Scott, 2008) in a new cultural context: the DOE in their exosystem.

Teachers mentioned that in order to obtain satisfactory scores on the ECERS and CLASS evaluations, it was necessary to comply with the DOE requirements and standards in the exosystem as well as to collaborate with DOE personnel, such as instructional coordinators and social workers who were in the teachers’ microsystem and mesosystem. A majority of our teachers indicated their confusion around different messages from instructional coordinators and social workers on ECERS and CLASS evaluations in the mesosystem, and therefore, how to prepare for them. Ms. Alicia expressed her experience about the confusion because the instructional coordinators and social workers, as well as the evaluators, lacked consensus which often ended against her belief—creating her own classroom environment and presenting it at her best:

The instructional coordinator and social worker need to get on the same page as ECERS reviewers in the classroom. They are all completely different. ECERS wants you to do this way, CLASS wants you to do this way, the DOE [instructional coordinator and social worker] wants you to put things away this way... For each one I have to change my room around. For each individual thing. What is the point of that?

Pre-K teachers were also required to attend 4 days of DOE sponsored PD sessions, and the programs at public schools are closed on those 4 days. However, it is not necessarily the case for several programs at CBOs because not all organizations follow the public school calendar. That is, the schedule variability between public schools and CBOs caused in the exosystem, potentially also resulting in the inconsistent expectations for teachers and complex relationships within their mesosystem. When these organizations need to stay open, teachers are unable to leave the classrooms unless site leaders find substitute teachers. Ms. Samantha described her missed PD opportunities:

I have never been to those PDs. It would have been nice to attend them. The DOE [needs to] look into the community-based organizations that they have [Pre-K] classrooms in, because now it’s part of the DOE system. Make sure that we’re still receiving the professional development support [from the DOE]. If you notice that a teacher doesn’t go to the PDs for the DOE all year, not really looking at what kind of support the teacher is receiving at her school… is it effective teacher support?

The teachers described their confusion around working with different DOE personnel, such as instructional coordinators and social workers in teachers’ microsystem, who sent different, or sometimes conflicting messages to teachers, possibly due to a lack of consensus around expectations in teachers’ mesosystems. This indicates that teachers felt the need to discontinue their own practice and shift their approaches to early childhood education to prioritize the requirements under PKFA at their microsystem level, which suggests that they continuously shifted their identity from moment to moment in various contexts.

### Theme 3: Having a brand new relationship with the DOE is hard (social nature)

Before the UPK expansion, the majority of CBO programs for 4-year-olds were funded through the ACS, not the DOE. Therefore, this expansion policy initiated brand new relationships with the DOE staff, standards, and regulations, which specializes in public school operations and academic outcomes. For the majority of our teachers, this was their first time partnering with the DOE; that is, a brand new entity in their exosystem since the UPK expansion started. For instance, they had to follow new requirements for curriculum development, assessment, family engagement, and program quality rating. In order for the DOE to help teachers enact these policy changes, they sponsored PD sessions and hired instructional coordinators, and intended to universally implement the instructional standards. The exosystem level change influenced who teachers work with in the microsystem and mesosystem levels. Ms. Heather said:

I hope the DOE looks into the organizations that they have classrooms in community-based organization… It is now part of them. It’s still part of the DOE system. Make sure that we are still receiving the professional development support.

While teachers needed to comply with the DOE requirements and expectations for curriculum, they also had a direct responsibility to maintain safe classrooms, uphold the program’s mission, and be responsive to the community needs. Teachers brought attention to some of the complications they experienced as they transitioned to working with the DOE, and how opportunities for professional learning were restricted by systemic barriers. Ms. Alicia said:

I just wish DOE was a little bit more involved [as much as ACS used to be] and making sure that no, when these are the days off, these are days off. When you’re supposed to go to PD workshops, you’re supposed to go to PD workshops… You’re supposed to do three field trips a year. Trips can start in January. Push for them!

Ms. Mabel explicitly demanded clearer messages from the DOE and expressed her disappointment in the way the CBO teachers were managed by the DOE system, especially the salary differential between the two school settings:

I think DOE needs to really, really check and see what it is they truly want and then let us know because they’re not clear. [...] The DOE also needs to pay teachers the same salary that they’re gonna pay the teachers in the public schools. That’s one of my big confusion with them, the requirements [to become a Pre-K teacher] are the same. You have to have the Master’s, you have to be certified. Why should you make ten, fifteen thousand dollars less [at CBOs] when you have to fulfill the same requirements? You still have the same loans that have to get paid back, so you should make the same money.

It was evident that CBO teachers had complicated relationships with the DOE at the time of interviews. The key players who interacted with teachers—e.g., instructional coordinators—encouraged them to do things the “DOE way” which ultimately shifted their practices to a more universal approach of PreK education.

The CBO teachers evidently found that the new relationships with the DOE were complicated as the teachers felt the strong need to comply with the DOE, but were kept untended and excluded from decision-making processes around their own teaching profession. Especially since Covid-19 pandemic started, teachers’ social relationships with children, families, administrators, and even the DOE had changed. Communications were done online, and classes were taught remotely. Teachers needed to navigate through the unknowns, support the families as well as their colleagues while they themselves needed much support pedagogically, physically, and mentally.

In sum, our findings indicate that the early childhood policy shift at teachers’ exosystem impacted other systems of their professional identity, mesosystem and microsystem.

## Discussion

Using the framework of the Ecological System of ECCE Teacher Professional Identity, this study explored the impact of universal Pre-K expansion policies on how teachers’ professional identity is shaped and reshaped in NYC through interactions among the teachers’ ecological systems. The findings shed light on how the Pre-K expansion influenced CBO teachers’ daily lives and practices in particular, through the integration of various agencies to their already multi-faceted ecological systems, namely the NYC DOE.

Three themes emerged in relation to teachers’ professional identity. First, becoming a teacher who can fulfill multiple roles to complete the added requirements and to meet DOE’s expectations during the Pre-K expansion was necessary for all of the teachers in this study. Second, continuously modifying or adjusting their own practices to meet DOE’s expectations and requirements was overwhelming for the majority of our teachers. Lastly, fostering a brand new relationship with the DOE was hard and teachers needed more clarity around how the new relationship would influence their daily operations.

The expansion, which was enacted in teachers’ exosystem, in time, resulted in added requirements, shifting expectations, and heightened accountability. The CBO teachers were already working under the systems of ACS, EarlyLearn, and in some cases, Head Start. The added and shifting expectations and requirements within the exosystem built on more complexity to teachers’ daily experiences, which required working with additional personnel such as instructional coordinators and social workers at the mesosystem and microsystem. Teachers needed to make adjustments to their practice and (re)shaped their professional identity. Moreover, as PKFA programming rolled out citywide, almost all other aspects of PKFA operations also seemed to happen universally, which included budgeting, quality improvement, learning assessment, kindergarten readiness, opportunities for teacher training and PD (NYC Department of Education, 2019). This was done in a top-down approach and resulted in heightened perceptions of compliance and teachers’ diminished autonomy. As program improvements are sought in ECCE settings across the U.S., all members of the early childhood ecological systems need to work together to exchange information and to engage with multiple perspectives from teachers to gain a deeper understanding on the impact of policy or practical recommendations to daily classroom life (Douglass, 2017).

Our interviews with teachers highlighted the issues around an equality focus that was embedded within the universalization at the exosystem level they encountered in meeting the increased expectations for quality programming. These disparities are fractures within the system related to CBO teachers’ PD, salary disparities, and an added requirement for credentialing. These perpetuate inequities in workforce development which is consistent with Wilinski’s (2017) findings. For instance, opportunities for professional learning and career ladders are secured for public school teachers by the DOE, yet these are not guaranteed for CBO teachers (Reid et al., 2018). The efforts to increase the number of certified professionals and elevate professional learning during Pre-K expansion can be transformative if there is greater attention paid to the structural issues that help or hinder teachers’ accessibility to resources and support. More importantly, the agencies within the exosystem can work towards equitable distribution of resources in order to meet the localized needs of teachers in various geographical neighborhoods, rather than universal distribution of such resources as budgeting, requirement for credentialing and continuing education, and opportunities for training and PD.

Most of the teachers in this study experienced the need to assimilate their professional identity in order to comply with the universal requirements and meet the universal expectations for UPK programs within the exosystem. They had to make more compromises to their own teaching practice, and were held accountable in the implementation process, as Ms. Jessica stated a few times, “doing it the DOE way” in their microsystem. Cohen (2010) discusses that teachers need a space for negotiation for their own professional identity development through exchanges within their collaborative relationships with other stakeholders. This suggests a more localized, individualized trajectory for development of professional identity that requires interactions among the exosystem. Supportive colleagueship as well as sensitive, purposeful, and more culturally relevant leadership within teachers’ mesosystem and microsystem, positively influence teachers’ professional identity and promote a sense of purpose, which can be a significant factor to enhance program quality rather than compliance (Day & Smethem, 2009). We argue that policymakers also approach policy changes with such supportive, sensitive, purposeful and culturally relevant leadership to help teachers continue developing healthy, positive professional identity, so every policy change does not have to force teachers to constantly assimilate their practices and become confused in their new roles and relationships.

In 2014, the number of Pre-K seats was steeply increased from 3000 to 30,000 in CBOs (NYC Independent Budget Office, 2015), and more than seventy percent of the teachers at CBOs were women of color in NYC (Reid et al., 2018). As mentioned earlier, early childhood teachers in local community organizations, that have historically served the communities in need, are predominantly people of color. The CBO teachers’ work conditions, compensations, and benefits differed from those of the teachers at public schools where more than 70% of the teachers were white (Reid et al., 2018). The reports on “universal” Pre-K programs illustrate that Pre-K teachers’ experiences greatly differ based on school contexts in which they teach: district schools or CBOs, and issues around equity and racial and socio-economic segregation in NYC schools still persist (Potter, 2016; Reid et al., 2018). These socio-cultural, political aspects in teachers’ chronosystem might have been integral factors that also influenced a teacher’s professional identity.

The policy enacted at a teacher’s exosystem level significantly affected their interactions and therefore relationships within their mesosystem and microsystem levels as well. All of which influenced shaping and reshaping CBO teachers’ professional identity in their new roles as early childhood educators working under a new entity, the DOE, during the PreK expansion.

Since the NYC UPK expansion started, CBO teachers have experienced various policy changes, and much focus has been on compliance and quality improvement. We argue that policymakers need to focus more on equitable, culturally relevant, and adaptive approaches to teachers, teacher education programs, and credentialing procedures that are also required to meet the various needs of teachers during the time of policy change. In order to foster such equitable learning and professional communities, all policymakers and stakeholders need to reflect on how CBO teachers’ practices have been influenced by policy change and what they need for culturally relevant teaching and PD. It is when those needs are met that teachers will start developing a healthy identity as early childhood professionals and actively make contributions to the society at large through their pedagogy. As Sachs (2003) notes, “teacher identity stands at the core of the teaching profession.”

The educational systems (e.g., the federal, state and local DOE, accrediting bodies such as the National Association for the Education of Young Children) set expectations around universal Pre-K programming and implementation, and teachers of color must learn ways to navigate these dominant systems in order for them to be considered “successful” in their profession. Some of these macrosystem level organizations have made efforts to address the systemic racial inequality that has long existed in education systems. For example, NYC DOE has required all Pre-K-12 teachers to take an implicit bias training since 2018. NAEYC, which exists in teachers’ macrosystem, has revised their position statement on Developmentally Appropriate Practice with a focus on advancing racial equity. Local level needs are great, yet teachers’ ‘funds of knowledge’ (Giraldo et al., 2017, p. 50; Moll et al., 1992), or resources such as lived experiences and knowledge of the social, cultural, and historical wisdoms of communities and cultures, are often clouded by structural barriers such as the pressure to comply across all levels of ecological systems. Policymakers need to generate a way to remove such structural and systemic barriers, including requiring implicit bias and racial equity PD to all teachers, critically re-considering the existing curricula to advance equity in classrooms and schools, and embrace the voices of teachers of color who hold the ‘funds of knowledge’ of the communities they serve.

For further analyses, researchers should consider the use of frameworks that may be utilized to address race and racism such as critical race theory (Bell, 1987; Harris, 1993) and the onto-epistemological diversities of early childhood (Peréz & Saavedra, 2017) to better understand the lived experiences of teachers of color in the systems that have been built on the dominant, White, euro-centric perspectives toward education. Furthermore, critical narrative analysis (Souto-Manning, 2014) allows researchers to examine narratives in the lives of people of color, i.e., ‘real-world issues’ (p. 163) within the context of socially constructed, institutional, power discourse (Souto-Manning & Cheruvu, 2016). We, as policymakers, policy enactors, and researchers who live and engage in the ecological systems of early childhood professions, need to understand CBO teachers’ experiences to critically develop policies that meet teachers’ needs and advance the quality of ECCE services in local neighborhoods, which was the original purpose of establishing community-based organizations.

A limitation of this study is the fairly small number of participants we had for the large number of PKFA lead teachers there are in NYC, which calculates roughly more than 4000 of them. Given that New York City is one of the most diverse cities in the United States, our participants do not reflect (or were not generalizable) those of the city. Therefore, a large racially diverse sample is needed for future studies.

## Conclusion

Pre-K for All initiative is in its 6th year, and it is more critical now than ever to deepen our understanding of the impact of policy change and reform on daily experiences of Pre-K teachers. In order to make these organizational efforts successful and sustainable, policymakers need to start valuing and listening to the voices of teachers at CBOs, to adopt policies to meet values, beliefs, experiences, and needs that shape practice and professional identity of early childhood teachers so a policy change does not have to force them to constantly assimilate and adjust who they are as professionals. The ECCE fields, including policymakers, policy enactors, and researchers, need to generate supportive, sensitive, purposeful, and culturally relevant leadership to develop support CBO teachers for closing the inequitable gap in work conditions, compensations, credentialing process, and benefits to stop perpetuating the existing structural barriers. Teachers also should have a voice in choosing what would be culturally relevant professional development opportunities that are helpful for them to develop their professional identity and to support their communities. We also need to develop our critical awareness, interrogate the long-held ‘normal’ in the ECCE field and take actions to create an equitable workforce where teachers form a healthy professional identity as early childhood educators. We must work in solidarity with CBO teachers and all other actors of early childhood care and education to remove structural barriers.

## Data Availability

Due to the nature of this research, participants of this study did not agree for their data to be shared publicly, so supporting data are not available.

## Abbreviations

NYC: New York City
UPK: Universal pre-kindergarten
ECCE: Early childhood care and education
PKFA: Pre-K for All
CBO: Community-based organization
PD: Professional development
ACS: Administration for Children’s Services
DOE: Department of Education
ECERS: Early Childhood Environmental Rating Scale
CLASS: Classroom Assessment Scoring System

## Footnotes

1 New early childhood funding system launched by the Mayor Bloomberg administration in 2012.

2 Since July 2019, the DOE supports and monitors all of child care and early childhood educational services for ages 0–5.

3 New York State requires a Bachelor’s degree for Initial Certificate, which is valid for 5 years. All teachers are required to hold a Professional Certificate to continue teaching within the public school system, for which completion of a Master’s degree is mandated.

4 Based on 37 weeks of operation in public schools and 52 weeks in CBOs, and 35 h of work in public schools and 45 h in CBOs (Reid et al., 2018).

5 MDRC determined resource levels by the concentration of poverty in community districts (low = more than 50% of the population; moderate = less than 30% of the population; high = less than 15% of the population).

6 In this study, we consciously chose the more inclusive terms Black and Brown to describe African Americans, Africans, Caribbeans, and South and Central Americans in NYC, where darker-skinned people are not only African Americans, but also those with foreign nationalities and recent immigrants and their families.

7 We used the term Latina as the teachers with Latin American background in this study self-identified as Latina.

8 Now it is known as The New York State Prekindergarten Learning Standards (2019).
